# Immunonutrition Changes Inflammatory Response in Colorectal Cancer: Results from a Pilot Randomized Clinical Trial

**DOI:** 10.3390/cancers13061444

**Published:** 2021-03-22

**Authors:** Mateusz Wierdak, Marcin Surmiak, Katarzyna Milian-Ciesielska, Mateusz Rubinkiewicz, Anna Rzepa, Michał Wysocki, Piotr Major, Stanisław Kłęk, Michał Pędziwiatr

**Affiliations:** 1 2nd Department of General Surgery, Jagiellonian University Medical College, 31-008 Krakow, Poland; mateusz.wierdak@uj.edu.pl (M.W.); mateusz.rubinkiewicz@uj.edu.pl (M.R.); a.rzepa@doctoral.uj.edu.pl (A.R.); m.wysocki@doctoral.uj.edu.pl (M.W.); piotr.major@uj.edu.pl (P.M.); 2 Department of Internal Medicine, Jagiellonian University Medical College, 31-008 Krakow, Poland; marcin.surmiak@uj.edu.pl; 3 Department of Pathomorphology, Jagiellonian University Medical College, 31-008 Krakow, Poland; katarzyna.milian-ciesielska@uj.edu.pl; 4 Surgical Oncology Clinic, National Cancer Institute, 31-501 Krakow, Poland; stanislaw.klek@onkologia.krakow.pl

**Keywords:** immunonutrition, colon cancer, inflammatory response, perioperative care, randomized controlled trial

## Abstract

**Simple Summary:**

Nutritional support for patients who underwent surgery for colorectal cancer is widely accepted for reducing the incidence of perioperative complications. Immunonutrition is generally recommended to decrease the incidence of infectious complications. However, there is little clinical data regarding the impact of such treatment on tumor biology. Some basic studies show its negative impact on the development of the tumor, while others suggest it might be beneficial. Currently, there is no clinical evidence for any effect of immunonutrition on tumor tissues in vivo. Therefore, we designed this pilot randomized controlled trial to investigate the impact of immunonutrition compared with standard nutritional support in the preoperative period on the inflammatory response, cytokine expression, and leukocyte infiltration in the tumor tissue. Changes in tumor necrosis factor alpha (TNF-α), interleukin 8 or chemokine (C-X-C motif) ligand (CXCL8), and chemokine (C-X-C motif) ligand 1 (CXCL1) expression were observed after the intervention. In the immune group, a decrease in neutrophil infiltration was observed. Immunonutrition in the preoperative period influenced inflammatory response in patients with colorectal cancer.

**Abstract:**

Introduction: Surgery is the first choice of treatment for colorectal cancer. Nutritional support in the form of oral nutritional supplements (ONSs) in the preoperative period is widely accepted for reducing the incidence of perioperative complications, and immunonutrition is generally recommended. However, there is little clinical data regarding the impact of such treatment on tumor biology. Material and Methods: In this study, tumor tissue and blood samples were collected from 26 patients during preoperative colonoscopy at the time of clinical diagnosis (sample A). Group 1 received standard ONSs (3× Nutricia Nutridrink Protein per day) for 2 weeks before surgery. In group 2, immune ONSs (2× Nestle Impact Oral) were administered for the same duration. Tumor tissue (sample B) was then retrieved from the tumor after resection. Changes in the expression levels of inflammatory cytokines (TNF-α, interleukin 8 or chemokine (C-X-C motif) ligand (CXCL8), stromal cell-derived factor 1 (SDF1a), chemokine (C-X-C motif) ligand 6 (CXCL6), chemokine (C-X-C motif) ligand (CXCL2), myeloperoxidase (MPO), and CXCL1) were assessed during the perioperative course. Results: TNF-α expression differed after intervention between the two groups (immune group 31.63 ± 13.28; control group 21.54 ± 6.84; *p* = 0.049) and prior to and after intervention in the control group (prior to intervention 35.68 ± 24.41; after intervention 21.54 ± 6.84; *p* = 0.038). Changes in CXCL8 expression in the control group occurred prior to and after intervention (prior to intervention 2975.93 ± 1484.04; after intervention 1584.85 ± 1659.84; *p* = 0.041). CXCL1 expression was increased in the immune group and decreased in the control group (immune group 2698.27 (1538.14–5124.70); control group 953.75 (457.85–1534.60); *p* = 0.032). In both groups, a decrease in superficial neutrophil infiltration was observed, but this was only statistically significant in the immune group. There was no impact of the observed differences between the two groups on surgical outcomes (morbidity, length of stay, readmissions). Conclusions: Immunonutrition in the preoperative period compared with standard nutritional support may influence inflammatory cytokine expression and leukocyte infiltration in patients with colorectal cancer.

## 1. Introduction

Surgery is the first choice of treatment for colorectal cancer. Nutritional support in the form of oral nutritional supplements (ONSs) in the preoperative period is widely accepted for reducing the incidence of perioperative complications [1,2]. Malnutrition is one of the main causes of postoperative complications in various cancer types [3]. Hence, the introduction of nutritional support in the perioperative period is recommended for malnourished patients or for those with high nutritional risk [2,4]. While the use of nutritional support to prevent or treat malnutrition in the preoperative period does not raise wider concerns, the use of immunonutrition (nutritional support containing components that interfere with the immune system: arginine, glutamine, omega-3 fatty acids, nucleotides, and zinc) is not widely accepted. The positive impact of immunonutrition compared with standard nutritional support in the reduction of postoperative infectious complications and other surgical outcomes has been reported [5,6]. However, this only refers to short-term clinical outcomes. There are many concerns about the potential impact of this treatment on tumor biology and neoplastic dissemination. Following several fundamental studies, several authors highlighted the potential negative impact of immunonutrition [7,8], while others suggested it might even be beneficial [9]. Currently, there is no clinical evidence for any effect of immunonutrition support on tumor tissues in vivo. Because of the noticeable benefits of immunonutrition in the perioperative period to reduce perioperative complications, determining the potential impact of such nutrition on tumor tissue is important. Therefore, we designed a randomized controlled trial to investigate the impact of immunonutrition compared with standard nutritional support in the preoperative period on the inflammatory response, cytokine expression, and leukocyte infiltration in the tumor tissue of patients who underwent surgery for colorectal cancer.

## 2. Materials and Methods

### 2.1. Patients

A single-center, randomized controlled, non-inferiority trial with two parallel intervention arms was conducted between November 2017 and November 2018 at a tertiary referral university hospital (Krakow, Poland). This trial was registered on clinicaltrials.gov (NCT04732442) after approval of the protocol by the local ethics committee.

### 2.2. Inclusion and Exclusion Criteria

This study recruited patients older than 18 years with a diagnosis of colorectal adenocarcinoma. Patients were randomly divided into two groups: group 1, which received perioperative oral nutritional supplements (ONSs) in the form of immunonutrition (immune group); and group 2, which was the control group and received standard ONSs preoperatively. Patients were enrolled after informed consent was obtained during preoperative counseling. All patients underwent an elective surgical procedure 21 to 28 days after qualification. The exclusion criteria were as follows: histopathological diagnosis other than adenocarcinoma, emergency/urgent surgery, active infection, history of inflammatory bowel disease, other systemic immune disorder, necessity of preoperative neoadjuvant treatment (radiotherapy or chemotherapy), metastatic disease, or local infiltration of cancer that was described as T4 stage in preoperative computed tomography (CT) scan. Patients who were not able to intake at least 85% of administered ONS doses were also excluded from the analysis.

### 2.3. Randomization

The 1:1 randomization with concealment was achieved using a random number generator (even/odd) [10]. Randomization was performed after primary colonoscopy, during which the location of the tumor was confirmed, and tumor tissue samples were taken for histopathological examination and molecular tests. During this procedure, blood samples were also taken for biochemical tests. The randomization process and assignment of the patients to the groups were performed by a trial researcher who was not directly involved in the surgery or perioperative care of the patients.

### 2.4. Study Protocol

This study included patients who were referred for surgical treatment due to colon cancer. During the first visit to the surgical clinic for qualification for treatment, before diagnostic colonoscopy, patients with a high suspicion of colorectal malignancy were informed about their proposed participation in a randomized controlled trial. Next, the patients were qualified for colonoscopy to verify the tumor location and to obtain histopathological confirmation of the diagnosis. Colonoscopy was performed several weeks before the planned surgery. During the colonoscopy and after visualization of the tumor, three samples were taken for histopathological examination and three samples for molecular analysis, in addition to the standard samples taken from the tumor tissue to assess the infiltration of the immune system cells. Additionally, blood samples were collected from all patients for biochemical measurements. All tissue and plasma samples were then immediately frozen at −80 °C and stored for further examination. After histopathological confirmation of adenocarcinoma, patients were scheduled for a follow-up visit. Patients’ demographics and possible Surgical Site Infection (SSI) risk factors, including age, sex, body mass index (BMI), smoking activity, preoperative immunosuppressive treatment, and incidence of co-morbidities, were prospectively collected. After clinical staging of the cancer, patients were randomly allocated to groups.

### 2.5. Intervention

The control group consisted of patients who, as part of the standard preoperative nutritional support, received standard protein ONS (3× Nutridrink Protein per day (Nutridrink Protein^®^ Nutricia; UK)) for 2 weeks before surgery. In the immune group, immunonutrition ONS (2× Impact Oral (Impact Oral^®^; Nestle, Switzerland)) was administered for the same duration. Patients were asked not to consume any other nutritional supplements or functional food. All patients were managed according to the Enhanced Recovery After Surgery (ERAS) Society perioperative care guidelines [2]. After 2 weeks, all patients underwent elective colon tumor resection. Surgical procedures were performed laparoscopically, as described previously [11]. Blood samples for biochemical tests were collected from the patients on the day of surgery. In all patients, the surgical specimen was dissected immediately in the operating theater, and tissue samples were collected. All patients were monitored for compliance with the recommendations of preoperative care and nutrition. Patients were excluded from further analyses if at least 85% of the preoperative care recommendations were not fulfilled or when less than 85% of the recommended ONS doses were taken.

### 2.6. Perioperative Care

All patients in the pre- and perioperative period were treated according to the ERAS protocol dedicated to colorectal surgery [2]. The same protocol was used as described in previous studies conducted at our center.

### 2.7. Tissue Tumor Cytokine Concentration Measurement

Tumor tissue samples collected during preoperative colonoscopy were immediately frozen and stored at −80 °C for further molecular analyses of cytokine expression, including tumor necrosis factor (TNF-α), interleukin 8 or chemokine (C-X-C motif) ligand (CXCL8), stromal cell-derived factor 1 (SDF1a), chemokine (C-X-C motif) ligand 6 (CXCL6), chemokine (C-X-C motif) ligand 2 (CXCL2), myeloperoxidase (MPO), and chemokine (C-X-C motif) ligand 1 (CXCL1). Immediately after removal of the tumor, the specimen was incised, and tissue samples were gathered and frozen as described above.

The serum concentration of cytokines was assessed using Luminex MagPlex Microsphere assays (Merck, Burlington, MA, USA) and the Luminex MAGPIX System (Luminex Corp., Austin, TX, USA). Results were calculated from the calibration curves and expressed as pg/100 µg of total protein, according to the manufacturer’s protocol, as described previously [12].

### 2.8. Tissue Tumor Neutrophile Infiltration Assessment

Tumor tissue samples collected during preoperative colonoscopy were stored in 10% buffered formalin solution and immediately sent for histopathological examination. The same procedure was repeated during the final surgery. During histopathological examination, the neutrophil infiltration in the superficial and deep (100 µm under the epithelial surface) layers was assessed. Neutrophil infiltration in the epithelial layer as well as in the stromal layer was separately assessed. Neutrophil infiltration was assessed by counting the number of neutrophils per 10 large microscopic fields.

### 2.9. End Point Criteria

#### 2.9.1. Primary Outcome

The primary outcome was the change in the expression of inflammatory cytokines (TNF-α, CXCL8, SDF1a, CXCL6, CXCL2, MPO, and CXCL1) after preoperative nutritional intervention in tumor tissue samples obtained prior to and after intervention.

#### 2.9.2. Secondary Outcomes

The secondary outcome was the change in tissue neutrophil infiltration after preoperative nutritional intervention in tumor tissue samples obtained prior to and after intervention.

### 2.10. Sample Size Calculation

In our previous observations, TNF-α tissue concentration was 38 ± 20 pg/100 µg total protein in the control group. To demonstrate that immunonutrition increased the expression of TNF-α by 60%, a total sample size of 24 subjects was needed for an alpha value of 0.05 and 80% power. Thus, with expectations of omissions, a total sample size of 14 patients in each arm was sought.

### 2.11. Statistical Analysis

Continuous data are presented as means and standard deviations (SD) for normal distribution and as medians and inter-quartile ranges (IQR) if the distribution was not normal. Continuous variables were compared using the Mann–Whitney test, Wilcoxon signed-rank test, and Student’s *t*-test. Categorical variables were compared using the chi-squared test, including Yates’ correction or Fisher’s exact test when necessary. The level of significance was set at *p* < 0.05. Analyses were performed with Statistica 13.5 software (TIBCO Softwere, Palo Alto, CA, USA).

## 3. Results

**Figure 1:**
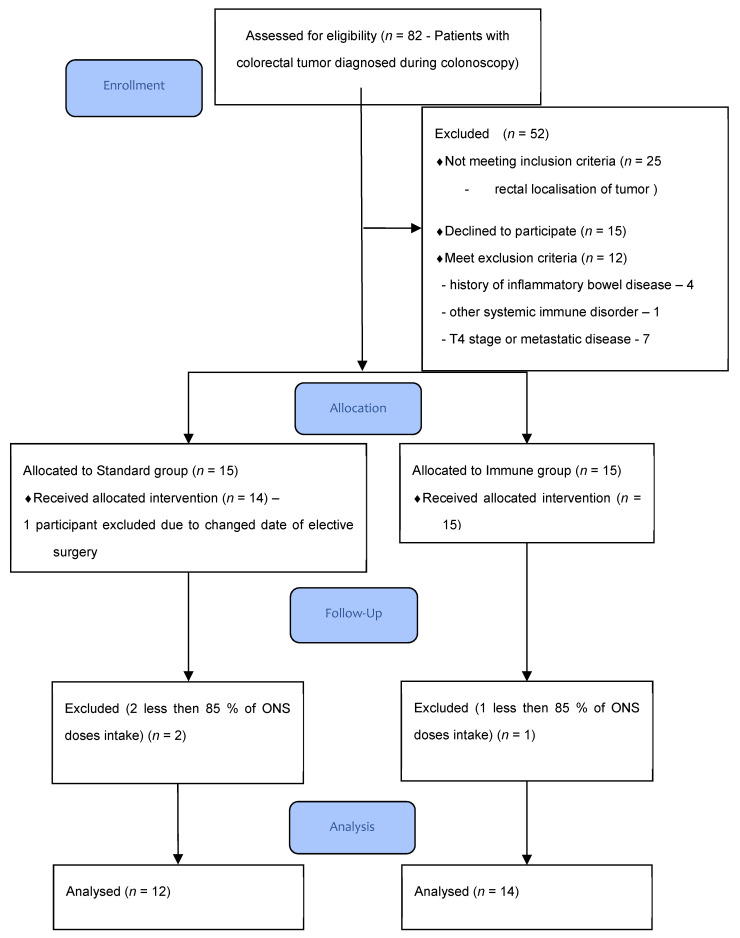
Patients flow-chart.

A total of 29 patients were randomized in the study. Three patients (10%) were lost to follow-up because less than 85% of the recommended ONS doses were taken. One patient was excluded after allocation due to a change in the planned surgery date. The patients’ flow through the study is presented in Figure 1.

Patients’ baseline characteristics are shown in Table 1, and blood parameters are shown in Table 2. Although an increase in the total protein and serum albumin was observed, which was greater in the immune group, it was not statistically significant. Differences in selected cytokines in tumor tissue before and after intervention are shown in Table 3. Differences were observed in TNF-α after intervention between the groups (immune group, 31.63 ± 13.28; control group, 21.54 ± 6.84; *p* = 0.049) and prior to and after intervention in the control group (prior to intervention, 35.68 ± 24.41; after intervention, 21.54 ± 6.84; *p* = 0.038). Changes in CXCL8 concentration in the control group were observed prior to and after intervention (prior to intervention, 2975.93 ± 1484.04; after intervention, 1584.85 ± 1659.84; *p* = 0.041). An increase in CXCL1 concentration in the immune group was observed, but a decrease was evident in the control group (immune group, 2698.27 (1538.14–5124.70); control group, 953.75 (457.85–1534.60); *p* = 0.032). Histopathological outcomes are presented in Table 4. There were no statistically significant differences in neutrophil infiltration between the two groups prior to intervention for any of the analyzed parameters. After intervention, changes in superficial neutrophil infiltration were observed between the groups. In both groups, a decrease in superficial neutrophil infiltration was observed, but this was only statistically significant in the immune group. There were differences in deep neutrophil infiltration before and after intervention in both groups, without any statistical significance. We did not observe any differences between the groups with respect to morbidity (immune group, 4 (28.5%); control group, 3 (25%); *p* = 0.9095), length of hospital stay (immune group, 5 [4,5,6,7,8,9,10,11,12,13,14,15]; control group, 5 [4,5,6,7,8,9] (median (IQR)); *p* = 0.8402) There was no mortality and only one readmission in the control group.

## 4. Discussion

The present clinical study showed that the use of immunonutrition in the preoperative period in colon cancer patients demonstrated not only the previously observed systemic effects [5] but also had a significant effect on the tumor tissue itself. This study is the first prospective randomized clinical trial to investigate this issue. Several studies previously analyzed the effect of immunonutrition on the clinical outcomes of colon cancer [6,13], but none have investigated the direct impact of this diet on tumor tissue.

The present study showed that both standard nutritional support and immunonutrition ONSs improved the nutritional status of patients in the preoperative period. Because of the nutritional intervention, none of the patients in the study exhibited clinical or laboratory symptoms of current or impending malnutrition. All patients had a BMI of >21.5 kg/m^2^. Only nine patients obtained a result of 3 or 4 in the preoperative assessment using the NRS 2002 scale, of which five were >70 years old (+1 point on the scale). Although increases in total protein and serum albumin were observed that were greater in the immunonutrition group, they were not statistically significant. This study analyzed a general population of patients with colon cancer. In this group, only a subset of the patients was malnourished, and only in these cases could we expect a significant improvement [14,15]. This increase was not as significant in other well-nourished individuals. We speculate that this is why the differences in protein and albumin levels prior to and after intervention were statistically significant. Our observations confirm the results of previous studies, which showed the beneficial effects of both immunonutrition and standard nutritional support in the preoperative period on the nutritional status of patients undergoing surgery for colorectal cancer [16,17].

No impact of the differences between the groups on surgical outcomes (morbidity, length of stay, readmissions, mortality) was observed. However, this study was not designed for such an analysis.

Chemokines, factors that stimulate the migration of cells of the immune system, may have a significant impact on the immune response to neoplastic cells [18,19,20]. Although basic research shows that lymphocytes (NK and Th1) have a major role in the immune surveillance of cancer cells, the regulation of their interactions is extremely complicated to analyze because of the divergence and convergence effects of various interleukins [21,22,23]. Therefore, in our study, we focused on assessing the chemokines that stimulate the migration of a wide range of immune system cells (mainly neutrophils) as a model for the overall stimulation of the immune response in tumor tissue. Our results showed that immunonutrition caused an increase in all analyzed chemokines (except CXCL3) and MPO; however, the obtained results did not reach statistical significance, most likely because of the insufficient number of patients. Nonetheless, what was most surprising in the standard diet group was that a downward trend was observed, which, in the case of TNF-α and CXCL8, was statistically significant. For TNF-α and CXCL1, these differences between the groups resulted in statistically significant outcomes. The primary role of TNF is in the regulation of immune cells. TNF, as an endogenous pyrogen, can induce fever, apoptotic cell death, cachexia, and inflammation, and inhibit tumorigenesis [24,25,26]. Thus, it can potentially play a crucial role in the inhibition of tumor spread. CXCL1 is produced by a variety of immune cells such as macrophages, neutrophils, and epithelial cells, or Th17 cell populations [27,28,29]. CXCL1 expression can also be indirectly induced by IL1, TNF-α, or IL17 produced by Th17 cells and is triggered mainly by the activation of NF-κB or C/EBPβ signaling pathways predominantly involved in inflammation, leading to the production of other inflammatory cytokines [27]. However, its role in cancer development is ambiguous. It stimulates cells of the immune system, but several studies have shown its stimulatory effects in the development of various tumors, including colorectal cancer, through its influence on the promotion of angiogenesis [30,31,32]. Similar controversies are associated with CXCL8. It is a critical mediator associated with inflammation, in which it plays a crucial role in neutrophil recruitment and degranulation [33]. However, CXCL8 has been implicated as having a role in colorectal cancer by acting as an autocrine growth factor in colon carcinoma cell lines as well as promoting cell division and possible migration by cleaving metalloproteinase molecules [34]. We did not observe an increase in CXCL8 in the immune group, and thus, we can conclude that the potentially stimulative effect of CXCL8 is not caused by immunonutrition.

Interestingly, there was an increase in MPO concentration in the immune group, which was not observed in the control group. Although the observed increase was not large enough to be statistically significant, it should be noted because MPO is most abundantly expressed in neutrophil granulocytes and produces hypochlorous acid in its antimicrobial function [35]. This may indicate that the increases in chemokines are mainly related to the stimulation of the neutrophil infiltration and not the stimulation of neoplastic tissue.

Because of the nature of the study, it was impossible to assess whether the observed differences had a significant clinical impact on the course of neoplastic disease treatment in such a small group of patients. Therefore, more research is required to confirm the observed results.

Although differences were noted in the histopathological examinations of the tumor specimens, they involved only a few parameters. Therefore, it is difficult to assess the actual impact of the observed change in the tissue with neoplastic infiltration.

The question also remains as to which of the immunonutrition components is responsible for the observed changes in chemokine levels, but arginine seemed to be the critical element. Arginine as a substrate for the synthesis of endogenous nitric oxide by inducible nitric oxide synthase (iNOS) may have an important role in the stimulation of both non-specific and specific inflammatory responses [9]. However, research revealed the dual role of iNOS in cancer tissue, which is strongly influenced by the cell situation and is environment-dependent. It may stimulate or inhibit tumor progression depending on the tumor type, as well as genetic changes and neoplastic cell differentiation [9].

In clinical studies, the role of arginine was confirmed in head and neck cancer patients, both in terms of the reduction in postoperative complications and long-term survival [36]. A similar effect may be the reason for the high effectiveness of arginine in colorectal cancer, as both of these neoplasms show high expression levels of argininosuccinate synthase (ASS1) [37]. However, this is not sufficiently supported by scientific evidence and requires further basic and clinical research.

The role of omega-3 unsaturated fatty acids in regulating the inflammatory response and cytokine production is also not fully understood. The modulatory effect on the inflammatory response associated with the use of this component in immunonutrition is most likely associated with its effects on the profile of the produced eicosanoids [38]. The changes in the concentration of omega-3 in relation to omega-6 in cell membranes affect the proportion of eicosanoids. Prostaglandins and leukotrienes have a much lower pro-inflammatory effect than omega-6 acids. It is postulated that this is the most important factor in the action of this element of immunonutrition [39]. A different profile of secreted eicosanoids may change the stimulatory path of lymphocytes and macrophages and thus alter the profile of the secreted cytokines as well as having an influence on the neutrophilic infiltration of tumor tissue. However, this has not been sufficiently analyzed in clinical trials thus far; therefore, our assumptions require validation in further studies.

Regarding the third component of the immunonutrition formula, nucleotides, the literature does not indicate their potential role in the secretion of cytokines. The postulated influence of nucleotides in immunonutrition is related to their relative deficiency during the severe inflammation caused by their excessive use by cells of the immune system. However, this effect does not seem to be important in our observations.

Clinical studies have demonstrated the positive impact of immunonutrition in head and neck cancer [40], gastric cancer [41], and colorectal cancer [6] patients. On the basis of these studies, a similar beneficial effect of immunonutrition is postulated for other solid tumors that require extensive surgery. Therefore, further studies on the influence of immunonutrition on tumor biology are required.

### The Limitations of the Study

There are several limitations of our study. Firstly, the differences of TNF-α level in this study and values assumed during the sample size calculation were observed. The post-hoc analysis revealed that the study for this primary outcome, despite obtaining statistically significant differences, achieved only 49%.

Because only one complex immunological product was compared, we could not determine which of the immunonutrition components had the biggest influence on the observed variability. Furthermore, this study included only patients with colorectal cancer, and thus our observations cannot be easily generalized to other cancer types. This study showed only the variability at the molecular and microscopic levels. We have no evidence that the observed changes had a significant clinical impact on the tumor biology and course of the neoplastic disease. The study was a single-center study, and therefore our findings should be validated in a larger group of patients in a multicenter study.

## 5. Conclusions

The use of immunonutrition in the preoperative period may influence an inflammatory response in colorectal tumor tissue compared with standard nutritional support. We observed differences in cytokine expression and neutrophilic infiltration intensity in tumor tissues following administration of immunonutrition in the preoperative period. Further studies should focus on the mechanism of this effect and the clinical impact of this treatment on the oncological and surgical outcomes of colon cancer patients to improve perioperative cancer care.

## Figures and Tables

**Table 1:** Demographic analysis of patient groups.

Parameter	Group 1 IMMUNE	Group 2 CONTROL	*p*-Value
Number of patients, *n*	14	12	-
Females, *n* (%)	7 (50.0%)	7 (58.3%)	0.6708
Males, *n* (%)	7 (50.0%)	5 (41.7%)	
Mean age, years ± SD	69.9 ± 10.9	68.4 ± 7.62	0.6908
Body mass index (BMI), kg/m^2^ ± SD	29.2 ± 5.5	27.8 ± 3.9	0.2565
ASA 1, *n* (%)	1 (7.1%)	1 (8.3%)	0.8402
ASA 2, *n* (%)	8 (57.1%)	8 (66.7%)	
ASA 3, *n* (%)	5 (35.7%)	3 (25.0%)	
Any comorbidity, *n* (%)	12 (85.7%)	8 (66.7%)	0.2504
Cardiovascular, *n* (%)	5 (35.7%)	3 (25.0%)	0.5551
Hypertension, *n* (%)	10 (71.4%)	7 (36.1%)	0.4849
Diabetes, *n* (%)	2(14.2%)	3 (25.0%)	0.4895
Renal disease, *n* (%)	1 (7.1%)	1 (8.3%)	0.7587
Other comorbidity, *n* (%)	2 (14.2%)	1 (8.3%)	0.9095
Smoking, *n* (%)	3 (21.4%)	2 (16.7%)	0.6357
AJCC Stage I, *n* (%)	3 (21.4%)	2 (16.7%)	0.4241
AJCC Stage II, *n* (%)	4 (28.6%)	6 (50.0%)	
AJCC Stage III, *n* (%)	6 (42.9%)	2 (16.7%)	
AJCC Stage IV, *n* (%)	1 (7.1%)	2 (16.7%)	
NRS 2000 median, (IQR)	2 (1–3)	2 (1–3)	0.7970
Tumor location			
Cecum, *n* (%)	3 (21.4%)	2 (16.7%)	0.7865
Ascending colon, *n* (%)	1 (7.1%)	3 (5.5%)	
Transvers colon *n* (%)	2 (14.2%)	1 (36.1%)	
Descending colon *n* (%)	1 (7.1%)	1 (11.1%)	
Sigmoid colon, *n* (%)	7 (50.0%)	5 (41.7%)	
Grading			
G1	3 (21.4%)	1(8.3%)	0.6533
G2	10(71.5%)	11(91.7%)	
G3	1 (7.1%)	-	

SD—Standard Deviation, ASA score—American Society of Anesthesiologists score, AJCC—American Joint Committee on Cancer, NRS—Nutrition Risk Screening, IQR—Interquartile range, G1-G3—Grading score 1–3.

**Table 2:** Blood parameters.

Parameter	Group 1 IMMUNE	Group 2 CONTROL	*p*-Value
Number of patients, *n*	14	12	-
Median WBC before intervention, 10^3^/mL (IQR)	6.60 (5.33–8.31)	8.11 (6.16–9.28)	0.1983
Median WBC after intervention, 10^3^/mL (IQR)	6.49 (5.59–8.96)	7.34 (6.06–8.15)	0.9350
*p*-value	0.2945	0.5751	
Median neutrophil before intervention, 10^3^/mL (IQR)	4.25 (5.40–2.15)	4.90 (3.20–5.80)	0.1063
Median neutrophil after intervention, 10^3^/mL (IQR)	3.80 (2.82–5.50)	4.72 (2.94–5.20)	0.7281
*p*-value	0.9165	0.9528	
Median lymphocytes before intervention, 10^3^/mL (IQR)	1.74 (1.57–2.47)	1.80 (1.53–2.46)	0.9128
Median lymphocytes after intervention, 10^3^/mL (IQR)	1.83 (1.50–2.60)	1.86 (1.56–2.44)	0.8167
*p*-value	0.4421	0.9528	
Median plasma protein before intervention, g/L (IQR)	65.4 (59.0–72.0)	68.0 (68.8–73.4)	0.4250
Median plasma protein after intervention, g/L (IQR)	69.5 (64.0–72.0)	67.5 (59.5–70.0)	0.3913
*p*-value	0.7221	0.4990	
Median plasma albumin before intervention, g/L (IQR)	38.6 (35.9–42.0)	42.0 (38.1–50,2)	0.9212
Median plasma albumin after intervention, g/L (IQR)	40.0 (35.0–44.7)	39.9 (35.6–43.0)	0.2390
*p*-value	0.8588	0.2626	

WBC—White Blood Cells.

**Table 3:** Comparison of differences of selected cytokines in tumor tissue concentration before and after intervention.

	Group 1—IMMUNE		*p*-Value	Group 2—CONTROL		*p*-Value	*p*-Value of Comparison of Changes in Parameters
TNF-α (pg/100 ug total protein) mean ± SD	27.79 ± 14.01	31.63 ± 13.28	0.551	35.68 ± 24.41	21.54 ± 6.84	0.038	0.049
CXCL8 (pg/100 ug total protein) mean ± SD	2608.87 ± 1715.15	2676.41 ± 1530.15	0.910	2975.93 ± 1484.04	1584.85 ± 1659.84	0.041	0.095
SDF-1a (pg/100 ug total protein) median (IQR)	399.94 (319.78–469.63)	469.63 (395.47–565.72)	0.477	421.01 (384.89–501.80)	358.68 (333.02–371.16)	0.823	0.205
CXCL6 (pg/100 ug total protein) median (IQR)	247.73 (109.53–467.97)	241.76 (155.02–372.85)	0.309	238.11 (103.41–371.02)	133.14 (92.60–197.79)	0.671	0.640
CXCL2 (pg/100 ug total protein) mean ± SD	625.63 ± 793.10	879.19 ± 1008.23	0.438	631.87 ± 570.80	301.03 ± 287.39	0.407	0.261
MPO (pg/100 ug total protein) mean ± SD	54,176.82 ± 36,077.57	63,096.97 ± 38,509.00	0.473	51,313.49 ± 21,340.86	51,114.38 ± 30,976.68	0.935	0.655
CXCL1 (pg/100 ug total protein) median (IQR)	1902.86 (1170.34–3517.76)	2698.27 (1538.14–5124.70)	0.821	2144.59 (808.68–5933.12)	953.75 (457.85–1534.60)	0.403	0.032

TNF-α—tumor necrosis factor, CXCL8—interleukin 8 or chemokine (C-X-C motif) ligand, SDF-1a—stromal cell-derived factor 1 also known as CXCL-12, CXCL6—chemokine (C-X-C motif) ligand 6, CXCL2—chemokine (C-X-C motif) ligand 2, MPO—myeloperoxidase, CXCL1—chemokine (C-X-C motif) ligand 1.

**Table 4:** Histopatological outcomes.

Parameter	Group 1 IMMUNE	Group 2 CONTROL	*p*-Value
Number of patients, *n*	14	12	-
Median superficial neutrophil infiltration before intervention, *n*/HPF (IQR)	47 (31.5–82)	61 (35–88)	0.5022
Median superficial neutrophil infiltration after intervention, *n*/HPF (IQR)	39 (31–57)	59 (50–86)	0.0033
*p*-value	0.2651	0.1709	
Median deep neutrophil infiltration before intervention, *n*/HPF (IQR)	51 (27.5–93.5)	54 (28–87)	0.9341
Median deep neutrophil infiltration after intervention, *n*/HPF (IQR)	36 (27–50)	37 (31–50)	0.7775
*p*-value	0.0865	0.6071	
Median change in combined superficial and deep neutrophil infiltration before and after intervention, *n*/HPF (IQR)	−21 (−80.5–68.5)	−5 (−45–64)	0.5458

HPF—high-power field.

## Data Availability

Data are available on demand. If needed, please contact the corresponding author.
